# Effectiveness, Safety and Patients’ Satisfaction of Nabiximols (Sativex^®^) on Multiple Sclerosis Spasticity and Related Symptoms in a Swiss Multicenter Study

**DOI:** 10.3390/jcm13102907

**Published:** 2024-05-14

**Authors:** Rosaria Sacco, Gianna Carla Riccitelli, Giulio Disanto, Julien Bogousslavsky, Ariane Cavelti, David Czell, Christian Philipp Kamm, Uta Kliesch, Simon Peter Ramseier, Claudio Gobbi, Chiara Zecca

**Affiliations:** 1 Multiple Sclerosis Center (MSC), Department of Neurology, Neurocenter of Southern Switzerland, 6900 Lugano, Switzerland; rosaria.sacco@eoc.ch (R.S.); giannacarla.riccitelli@eoc.ch (G.C.R.); giulio.disanto@eoc.ch (G.D.); claudio.gobbi@eoc.ch (C.G.); 2 Faculty of Biomedical Sciences, Università della Svizzera Italiana (USI), 6900 Lugano, Switzerland; 3 Neurocenter, Swiss Medical Network, Clinique Valmont, 1823 Montreux, Switzerland; jbogousslavsky@cliniquevalmont.ch; 4 Regionalspital Emmental, 3400 Burgdorf, Switzerland; ariane.cavelti@spital-emmental.ch; 5 NeuroMedics, Praxis Neurologie Uster, 8610 Uster, Switzerland; david.czell@hin.ch; 6 Luzerner Kantonsspitals (LUKS), 6000 Luzern, Switzerland; christian.kamm@luks.ch; 7 Neurologische Praxis Schwyz—Zug, 6430 Schwyz, Switzerland; uta.kliesch@hin.ch; 8 Neurobaden—Praxis für Neurologie, 5405 Baden, Switzerland; simon.ramseier@hin.ch

**Keywords:** multiple sclerosis, nabiximols, spasticity, gait, pain, urinary symptoms

## Abstract

**Background:** Cannabinoid oro-mucosal spray nabiximols is approved for patients with moderate to severe multiple sclerosis spasticity (MSS) resistant to other antispastic medications. Few real-world data are available on the effectiveness, safety and patients’ satisfaction in MS patients treated with nabiximols as monotherapy. **Methods:** To investigate the effectiveness, tolerability and satisfaction of nabiximols in a real-life multicentric Swiss cohort as monotherapy or with stable doses of other antispastic medications, and explore clinical features which may predict treatment response. The following data were collected at treatment start (baseline) and 12 weeks thereafter: Modified Ashworth scale (MAS), scores at numerical rating scales ranging from 0 (absent) to 10 (considerable) for effect on spasticity (sNRS), pain (pNRS), gait (gNRS), urinary symptoms (uNRS), tolerability (tNRS) as assessed by the treating neurologist, and overall treatment satisfaction (TsNRS) and tolerability (tNRS) as assessed by the patient. **Results:** Ninety-five patients (44 relapsing remitting, 37 secondary progressive and 14 primary progressive MS; median age = 53 (IQR 45–62); female 70%; median EDSS 6 (IQR 4–6), concomitant antispastic treatments in 54% of patients) were included. From baseline to week 12, median MAS score decreased from 3.0 to 2.0 (*p* < 0.001). Median scores of the each NRS also significantly decreased (*p* < 0.001 for all comparisons). At week 12, the median TsNRS and tTS scores were 8/10 (IQR: 6–9) and 9/10 (IQR: 7–10), respectively, and 93.7% of patients continued to use nabiximols at the average dose of six sprays/day. No clinical factors, including use of nabiximols as add on vs. monotherapy, were associated with responder status. **Conclusions:** Our first Swiss, multicentric, observational, real-life study supports and enhances previous finding of nabiximols as monotherapy and as add-on therapy, being an effective, safe and well-tolerated treatment option for resistant MS spasticity and spasticity-related symptoms (pain, bladder dysfunction and gait).

## 1. Introduction

Spasticity is one of the most common and disabling symptoms of multiple sclerosis (MS), affecting up to 80% of patients, tending to worsen over time with moderate to severe spasticity involving one third of patients after 10 years from diagnosis [1,2,3]. Spasticity and associated symptoms, such as spasms, pain, sleep disturbances, and bladder dysfunction, interfere with patients’ mobility and daily activities, impairing autonomy and quality of life [4,5], and increasing healthcare burden [3].

MS spasticity management varies across countries, often involving a multidisciplinary approach [6]. First line treatments are physiotherapy and pharmacotherapy including baclofen, tizanidine, dantrolene, gabapentin, clonazepam, and botulin toxin A, although the quality of evidence supporting the use of these drugs is low or even lacking [7]. Additionally, these treatments are not effective or poorly tolerated in a significant proportion of patients, resulting in inadequate symptom management [8]. Another treatment option is represented by the intrathecal baclofen subcutaneous pump, limited however by surgical/post-surgical risks and the need to turn off the pump for performing an MRI [9].

Nabiximols is a complex botanical mixture containing balanced quantities of Δ-9-tetrahydrocannabinol (THC) and cannabidiol (CBD), along with other cannabinoid and non-cannabinoid components [10,11,12].

A number of randomized controlled trials [13,14,15] exploring the efficacy and safety of nabiximols mainly as add-on therapy in MS patients have reported a significant reduction in spasticity compared with placebo, a rapid occurrence of treatment response (within the firsts 4 weeks), a persistent efficacy over time without need to increase the dosage, and a low drug discontinuation due to side effects or inefficacy (10%) [13].

In 2013, nabiximols oro-mucosal spray [Sativex^®^, Almirall, Alte Winterthurerstrasse 14, 8304 Wallisellen (CH), Switzerland] was approved in Switzerland as treatment for MS patients with moderate to severe spasticity inadequately responsive to other antispastic medications [10,11]. 

Given the absence of a national Swiss registry on the use of nabiximols, we conducted a post-marketing survey in Swiss MS patients to collect data on real-world effectiveness, tolerability and satisfaction of nabiximols used as add on treatment or monotherapy for spasticity, and to identify clinical factors which may predict treatment response.

## 2. Materials and Methods

This was a prospective, observational, multicenter Swiss study conducted between July 2020 and June 2021 collecting data on effectiveness, tolerability, safety and patient’s satisfaction related to the use of sabiximols oro-mucosal spray in the treatment of spasticity, pain and other spasticity related symptoms in MS patients. 

Patients affected with MS according to McDonald 2017 [16] were recruited during routine neurologic visits upon decision to start a treatment with nabiximols. Exclusion criteria for nabiximols treatment were severe cardiovascular diseases, history of psychiatric diseases, and use of street cannabis and/or other psychoactive drugs. All patients signed an informed consent for the use of their clinical data. 

Demographic and clinical data on patients’ age, gender, MS course, and disease duration, were collected at baseline and 12 weeks after starting nabiximols. A complete neurological examination, including evaluation of disability by Expanded Disability Status Scale (EDSS) [17] and spasticity by Modified Ashworth scale (MAS) [18], was performed by the same neurologist at baseline and after 12 weeks, in order to reduce inter-rater variability. Nabiximols was initiated as a single spray on the evening of the first day of treatment and was gradually titrated by one additional spray every two days until the 4th day and then by one additional spray per day according to a prespecified dose escalation protocol (swissmedicinfo.ch) until patients experienced acceptable spasticity and pain relief, unacceptable side effects, or reached the maximum allowed daily dosage of 10 sprays per day. Patients were also asked to complete the self-reported scales for spasticity (Numerical Rating Scale for spasticity [sNRS]), spasticity related symptoms as pain (pNRS), urinary disorders (uNRS), gait impairment (gNRS), treatment satisfaction (tsNRS) and tolerability (tNRS) [19].

The Modified Ashworth Scale (MAS) is the most widely used clinical scale used to measure the increase of muscle tone. [20] It is a 6-point scale for assessing the degree of spasticity, from 0 referring to “no increase in muscle tone” to 5 referring to “limb is rigid in flexion or extension” [20]. The maximum MAS Score in this study was 5.

sNRS is a self-reported scale scoring from 0 (no spasticity) to 10 (worst possible spasticity) widely used to assess MS spasticity evolution during treatment with nabiximols [19]. For the evaluation of spasticity-related symptoms the pNRS, uNRS, and gNRS, scoring from 0 (no symptoms) to 10 (the worst symptoms), were used [19]. Treatment satisfaction and tolerability were investigated at 12 weeks using treatment satisfaction NRS (tsNRS) scoring from 0 (not satisfied at all) to 10 (very satisfied), and tolerability NRS (tNRS) scoring from 0 (lowest tolerability) to 10 (highest tolerability) [19]. 

Patients were defined as responders to nabiximols in case of an improvement of at least 30% in the sNRS score after 12 weeks of treatment [19,21]. In a further analysis, we defined nabiximols responders as those patients improving by at least 30% in the pNRS score after 12 weeks of treatment, pain being the most frequent and invalidating spasticity-related symptom [8].

New neurological symptoms and adverse events were registered during each follow-up visit.

The aims of our study were to: (a) investigate the effectiveness, tolerability and satisfaction of nabiximols in a real-life multicentric Swiss cohort of MS patients as monotherapy or with a stable doses of other antispastic medications; (b) explore whether clinical features may predict treatment response. 

### Statistics

All continuous variables are reported as median values and interquartile range (IQR), and categorical variables as numbers and percentages. Statistical significance level was considered to be 95% CI (two-sided alpha *p* < 0.05). Differences in demographic and clinical features between treatment responders and non-responders was assessed using Mann–Whitney U test on continuous variables, and the chi-square test or Fisher’s exact test on categorical variables. To investigate the relationship between demographic and clinical features and the effect of treatment, we used univariate and multivariable logistic regression models including age, gender, disease duration, EDSS, concomitant use of physiotherapy, concomitant pharmacological treatments for spasticity and use of disease-modifying therapies. *p* values < 0.05 were considered to indicate statistical significance. IBM SPSS Statistics 24.0 software was used to conduct data analyses.

## 3. Results

### 3.1. Baseline Characteristics

Ninety-five MS patients were included. Median (IQR) age was 53 (45–62) years, median disease duration 17 (9–26) years. The majority of patients were affected with progressive MS (14 patients [15%] with primary progressive and 37 [39%] with secondary progressive), while 44 [ (46%] patients had relapsing remitting MS. Median (IQR) EDSS was 6 (4–6). Sixty-six (70%) patients were receiving a disease modifying therapy for MS, 54 (57%) one or more other symptomatic treatments for spasticity and 89 (94%) physiotherapy (Table 1).

### 3.2. Spasticity Course before and after Treatment—Overall Population

After titration, the median number of daily nabiximols doses was 6 (IQR 4–8). Before treatment, the median spasticity severity as measured by MAS score was 3 (IQR 2–4) and decreased to 2 (IQR 1–3) at 12-week follow-up (*p* < 0.001).

At baseline, median sNRS was 7 (IQR 5–8) and improved to 5 (IQR 2–7) 12 weeks after treatment start (*p* < 0.001). Similarly, pNRS, gNRS and uNRS were 7 (IQR 4–8), 7 (IQR 2–7) and 5 (IQR 2–7) at baseline, while respective values decreased to 3 (IQR 2–5), 6 (IQR 4–8) and 3 (IQR 2–6) after 12 weeks of treatment (*p* < 0.001 for all comparisons).

### 3.3. Treatment Responders (TR) and Treatment Non-Responders (TNR) 

Twelve weeks after nabiximols start, 46 (48%) patients improved by at least 30% at sNRS score and were classified as treatment responders (TR), while the remaining 49 (52%) were classified as treatment non-responders (TNR). Median number of daily nabiximols doses was similar in TR and TNR (6 [IQR 3–8] vs. 5 [IQR 0–8], *p* = 0.292). TR showed a significantly lower median sNRS (3 [2,3,4] vs. 6 [5,6,7,8], *p* < 0.001), pNRS (2 [2,3,4] vs. 5 [3,4,5,6,7], *p* < 0.001), gNRS (4 [2,3,4,5,6,7,8] vs. 7 [5,6,7,8,9], *p* = 0.01), and uNRS (3 [1,2,3,4,5] vs. 4 [2,3,4,5,6], *p* = 0.05) when compared to TNR. 

Univariate linear regression analysis investigating possible predictors of nabiximols response showed an association between treatment responder status and EDSS score (OR = 0.73, 95% CI: 0.57–0.92; *p* = 0.01). Instead, the concomitant use of other pharmacological treatments for spasticity, age, gender, disease duration, concomitant use of physiotherapy, and use of disease-modifying therapies were not associated with responder status. No associations were found in the multivariable model (Table 2).

In a further analysis focusing on spasticity-related pain, we found an improvement of at least 30% in pNRS score at 12 weeks of treatment to be associated with EDSS score (OR = 0.62, 95% CI: 0.48–0.81; *p* < 0.001), disease duration (OR = 0.95, 95% CI: 0.912–0.990; *p* = 0.015), and concomitant use of other pharmacological treatments for spasticity (OR = 0.38, 95% CI: 0.16–0.94; *p* = 0.035) at the univariate model. However, EDSS score only maintained association at the multivariable model (OR = 0.68, 95% CI: 0.51–0.91; *p* = 0.008) (Supplementary Table S1).

### 3.4. Safety and Tolerability 

Six (6.3%) out of 95 patients discontinued nabiximols between baseline and week 12 because of side effects (N = 5) or insufficient efficacy (N = 1). The most common adverse events were dizziness (N = 5), confusion/ideomotor slowing (N = 2), fatigue (N = 1) and pharyngeal irritation (N = 1). Median (IQR) subjective tolerability to nabiximols rated at tNRS scale was 9 (7–10). Overall, patients were satisfied with the treatment (median tsNRS 8 (6–9)).

## 4. Discussion 

Efficacy and safety of nabiximols have been demonstrated in several clinical trials [13,15,22,23,24] and post-marketing observational studies, particularly as an add on treatment [25,26,27,28,29]. To date, this is the first real-world study investigating the use of nabiximols in an MS population that includes a large proportion (43%) of subjects receiving nabiximols as a monotherapy against spasticity and related symptoms. 

Firstly, by using the same definition of clinically relevant responder status and the same clinical scores, we found a higher proportion of responders to nabiximols (48%) compared with pre-approval clinical trials (36–40%) [15,23] and post-marketing observational studies (30–41%) probably due to different populations and time windows for responder-status evaluation. Actually, previous studies required patients to qualify as early responders, defined as a ≥20% sNRS improvement at 4 weeks, in order to continue nabiximols treatment and be evaluated as a ≥30% sNRS improvement responders at a later time point of 12 weeks [25,26,30,31]. In contrast, we observed all patients until 12 weeks when responder status was evaluated as ≥30% sNRS improvement. A single Belgian retrospective study found a higher number of treatment responders (74%), possibly confounded by recall bias [32]. 

Treatment response was consistent among all outcome measures, as responders to nabiximols also had significantly improved spasticity-related symptoms including pain, as well as spasticity-related urinary and gait problems, compared to non-responders [13,15,22,23].

In line with the registration clinical trial [13,15,22,23,33,34] and associated extension studies [33,34,35], discontinuation rate in our study was low (5%) and mainly due to dizziness and fatigue. Accordingly, our patients showed a high treatment satisfaction and tolerability scores. However, various side effects have previously been described in the course of nabiximols therapy. Among these, the most frequent are dizziness and fatigue, followed by dry mouth, balance impairment, nausea, headache, oral pain, somnolence, confusion, depressed mood, constipation, disorientation, dysgeusia, disturbance in attention, euphoric mood, vision blurred, and weakness [10].

Importantly, we conducted an analysis to identify patients’ baseline features predicting treatment response. We found that lower EDSS was associated with higher probability of responding to nabiximols. While this was not confirmed after weighting for other variables, lower EDSS was consistently associated with a reduction of at least 30% in spasticity associated pain. Instead, a post hoc analysis of the SAVANT study showed a reduction in sNRS and pNRS under nabiximols treatment irrespective of patients’ baseline characteristics, including EDSS [36]. This discrepancy might be related to differences in patients’ characteristics across studies, and to the observation by Carotenuto et al. that less disabled MS patients are less prone to develop adverse events and discontinue nabiximols in real world clinical practice [37].

Intriguingly, we did not find evidence of an association between responder status and the use of nabiximols either as monotherapy or add-on treatment. This suggests that monotherapy with nabiximols is effective against spasticity at least in some patients. Clinicians could therefore evaluate the opportunity to taper or discontinue other symptomatic therapies for spasticity in selected patients once nabiximols has been started with beneficial effects.

Our study has three main limitations. The first is the small sample size. A larger sample size might have perhaps allowed detection of different responses to nabiximols in patients treated with high effective disease modifyng therapies (DMTs), those treated with low effective DMTs and patients untreated. The second limitation is the use of a patient-reported outcomes (PROs) measure, as sNRS, to assess the nabiximols response. The improvement in sNRS may be partially due to a nabiximols effect on spasticity-related symptoms, including pain, as well as spasticity-related urinary and gait problems. However, sNRS was found to be both a reliable and valid scale for clinical evaluation of spasticity [19]. The third limitation is the short follow-up. Nonetheless, after ten years of nabiximols approval, ours is the first Swiss observational, prospective study showing effectiveness, safety and tolerability of nabiximols used as monotherapy or add-on therapy for treating MS spasticity and related symptoms. 

Future international multicenter real-world prospective studies should be performed to investigate the efficacy of nabiximols monotherapy on spasticity and spasticity-related symptoms during a long-term follow-up, as well as to explore the clinical and demographic features predictive of responder status. 

## Figures and Tables

**Table 1:** Baseline characteristics of patients, overall and stratified according to responder status.

	Overall	Treatment Responders	Treatment Non-Responders	*p*
	N = 95	N = 46	N = 49	
**Age** (years)				
- Median	53	50.5	56	0.048
- IQR	45–62	42–59	48–65	
- Range	25–86	30	26–77	
**Gender**				
- F	66 (70%)	30 (65%)	36 (74%)	0.504
- M	29 (30%)	16 (35%)	13 (26%)	
**Disease duration (years)**				
- Median				
- IQR	17	16	20	0.254
- Range	9.0–26	9–22	8–27	
	2–36	3–35	1–40	
**MS course (N,%)**				
- RRMS	44 (46)	26 (57)	18 (37)	0.246
- SPMS	37 (39)	16 (35)	21 (43)	
- PPMS	14 (15)	4 (9)	10 (20)	
**EDSS (median, IQR)**	6.0 (4.0–6.0)	4.0 (3.0–6.0)	6.0 (4.0–6.0)	0.006
**DMT (N,%)**				
- No	29 (30)	12 (26)	17 (35)	0.383
- Yes	66 (70)	34 (74)	32 (65)	
**Ashworth score**				
- Median	3	3	3	0.005
- IQR	2–4	2–3	2–4	
- Range	1–5	1–5	1.5	
**Current physiotherapy (N, %)**				
- No	6 (6)	3 (7)	3 (6)	
- Yes	89 (94)	43 (93)	46 (94)	1.000
**Current drugs for spasticity**				
- No	41 (43%)	24 (52%)	17 (35%)	
- Yes	54 (57%)	22 (48%)	32 (65%)	
-	**N = 54**	**N = 22**	**N = 32**	
- 1	43 (80%)	17 (77%)	26 (53.1)	
- >=2	11 (12%)	5 (23%)	6 (12.2)	
	**N = 54**	**N = 22**	**N = 32**	
- Tizanidine	25 (46%)	9 (41%)	16 (50%)	
- Tolperisone	2 (4%)	1 (5%)	1 (3%)	
- Baclofene	28 (52%)	11 (50%)	17 (53%)	
- Antiepileptics	9 (17%)	4 (18%)	5 (16%)	
- Others	10 (19%)	7 (32%)	3 (9%)	

**Table 2:** Regression models investigating associations between clinical characteristics and improvement by at least 30% in sNRS after 12 weeks of treatment.

Independent Variables	B	OR	95% C.I. for OR	*p*
Univariate analysis				
Age (per year of age)	−0.031	0.969	0.937–1.003	0.072
Sex (female vs. male)	0.390	1.477	0.614–3.553	0.384
Disease duration	−0.025	0.975	0.938–1.014	0.204
EDSS	−0.319	0.727	0.571–0.925	0.010
Physiotherapy	−0.067	0.935	0.179–4.884	0.936
Disease modifying treatments	0.409	1.505	0.623–3.639	0.364
Other spasmolytic drugs	−0.756	0.469	0.198–1.111	0.085
**Multivariable analysis**				
Age (per year of age)	−0.018	0.983	0.942–1.025	0.413
Sex (female vs. male)	0.274	1.315	0.486–3554	0.590
Disease duration	0.001	1.001	0.954–1.050	0.982
EDSS	−0.247	0.781	0.578–1.056	0.109
Physiotherapy	0.723	2.060	0.301–14.106	0.462
Disease modifying treatments	−0.041	0.960	0.338–2.726	0.939
Other spasmolytic drugs	0.705	2.024	0.767–5.342	0.154

B: Coefficient; C.I.: Confidence interval; OR: odds ratio; *p*: Significant ≤ 0.05.

## Data Availability

The data presented in this study are available on reasonable request from the corresponding author.

## Supplementary Materials

The following supporting information can be downloaded at: https://www.mdpi.com/article/10.3390/jcm13102907/s1, Table S1: Logistic regression models.
