# Supplementary material for: Effectiveness, Safety and Patients’ Satisfaction of Nabiximols (Sativex®) on Multiple Sclerosis Spasticity and Related Symptoms in a Swiss Multicenter Study
Source: J Clin Med. 2024 May 14;13(10):2907. doi: 10.3390/jcm13102907 (PMC11122311; doi:10.3390/jcm13102907)
Supplement: Supplementary file 1 [file jcm-13-02907-s001.zip › jcm-2995175-supplementary.pdf]

Journal of Clinical Medicine

MDPI

SUPPLEMENTARY TABLE S1. **Logistic regression models**

**Independent variables**	**B**	**OR**	**95% C.I. for OR**	**P**
**Univariate analysis**				
Age (per year of age)	-0.019	0.981	0.949-1.014	0.256
Sex (female vs male)	0.642	1.900	0.769-04.697	0.165
Disease duration	-0.051	0.950	0.912-0.990	**0.015**
EDSS	-0.471	0.624	0.480-0.812	**<0.001**
Physiotherapy	-1.497	0.224	0.025-1.994	0.180
Disease modifying treatments	0.575	1.778	0.737-4.291	0.201
Other spasmolytic drugs	-0.962	0.382	0.156-0.935	**0.035**
**Multivariate analysis**				
Disease Duration	-0.026	0.975	0.931-1.021	0.279
Other spasmolytic drugs	0.686	1.986	0.754-5.233	0.165
EDSS	-0.385	0.680	0.511-0.905	**0.008**

Regression models investigating associations between clinical characteristics and improvement by at least 30% in pNRS after 12 weeks of treatment. B: Coefficient; C.I.: Confidence interval; OR: odds ratio; p: Significant ≤0.05.
